# A scoping review of intimate partner violence assistance programmes within health care settings

**DOI:** 10.1080/20008198.2017.1314159

**Published:** 2017-05-05

**Authors:** Sheila Sprague, Taryn Scott, Alisha Garibaldi, Sofia Bzovsky, Gerard P. Slobogean, Paula McKay, Hayley Spurr, Erika Arseneau, Muzammil Memon, Mohit Bhandari, Aparna Swaminathan

**Affiliations:** ^a^ Division of Orthopaedic Surgery, Department of Surgery, McMaster University, Hamilton, Canada; ^b^ Department of Health Research Methods, Evidence, and Impact, McMaster University, Hamilton, Canada; ^c^ Department of Orthopaedics, University of Maryland School of Medicine, Baltimore, MD, USA; ^d^ Graduate Entry Medicine, Royal College of Surgeons in Ireland, Dublin 2, Ireland; ^e^ Department of Family Medicine, McMaster University, David Braley Health Sciences Centre, Hamilton, Canada

**Keywords:** Domestic violence, woman abuse, violence against women, intimate partner abuse, spouse abuse

## Abstract

**Background**: The lifetime prevalence of intimate partner violence (IPV) for women presenting to health care settings is estimated to be 38–59%. With the goal of providing help to victims of abuse, numerous IPV assistance programmes have been developed and evaluated across multiple health care settings.

**Objective**: Our scoping review provides an overview of this literature to identify key areas for potential evidence-based recommendations and to focus research priorities.

**Methods**: We conducted a search of MEDLINE, Embase, Cumulative Index of Nursing and Allied Health Literature, Cochrane Database of Systematic Reviews, Cochrane Central Register of Controlled Trials, and psycINFO. We used broad eligibility criteria to identify studies that evaluated the effectiveness of IPV assistance programmes delivered within health care settings. We completed all screening and data extraction independently and in duplicate. We used descriptive statistics to summarize all data.

**Results**: Forty-three studies met all eligibility criteria and were included in our scoping review. Nine categories of assistance programmes were identified: counselling/advocacy, safety assessment/planning, referral, providing IPV resources, home visitation, case management, videos, provider cueing, and system changes. Characteristics of programmes amongst studies frequently reporting positive results included those in which one type of active assistance was used (77.8% of studies reported positive results), a counsellor, community worker, or case manager provided the intervention (83.3% of studies reported positive results), and programmes that were delivered over more than five sessions (100.0% of studies reported positive results).

**Conclusions**: IPV assistance programmes are heterogeneous with regards to the types of assistance they include and how they are delivered and evaluated. This heterogeneity creates challenges in identifying which IPV assistance programmes, and which aspects of these programmes, are effective. However, it appears that many different types of IPV assistance programmes can have positive impacts on women.

## 1. Background

Intimate partner violence (IPV), also known as domestic violence, is a serious public health problem that results in substantial morbidity and mortality (Centers for Disease Control and Prevention, 2015). Globally, one out of every three women is physically or sexually assaulted by an intimate partner (World Health Organization, 2016) and two-thirds of the victims of homicides committed by intimate partners or family members are women (United Nations Office on Drugs and Crime, 2014). Victims of IPV experience more physical and mental health problems (Centers for Disease Control and Prevention, 2008; Coker et al., 2002; Ellsberg, Jansen, Heise, Watts, & Garcia-Moreno, 2008) and visit health care professionals (HCPs) more frequently, resulting in greater health care resource usage (Gass, Stein, Williams, & Seedat, 2010; Rivara et al., 2007). Previous research has found that the estimated lifetime IPV prevalence for women presenting to HCPs is 38–59% (Sprague et al., 2014), suggesting that the prevalence of IPV amongst women presenting to HCPs is higher than that of the general population.

HCPs are well positioned to identify and provide assistance to women experiencing IPV. The World Health Organization (2013) recommends that health care professionals offer intervention to all women who disclose IPV victimization. This support should include psychological support and validation as well as assistance with accessing resources and increasing safety. Previous qualitative and mixed method research has found that women generally report positive experiences when IPV interventions are initiated by their healthcare providers (Joyner & Mash, 2011; Prosman, Lo Fo Wong, Römkens, & Lagro-Janssen, 2014; Rees, Zweigenthal, & Joyner, 2014). Many different IPV assistance programmes have been developed and evaluated across multiple health care settings, however, variation in programme content and settings as well as the heterogeneity in research methodology and outcome measures have created challenges in determining the optimal assistance intervention (Bair-Merritt et al., 2014; Jahanfar, Howard, & Medley, 2014; Van Parys, Verhamme, Temmerman, Verstraelen, & Vermund, 2014). The purpose of our scoping review is to identify and provide an overview of the available literature evaluating IPV assistance programmes for women within health care settings to identify key areas for potential evidence-based recommendations and focus research priorities in the field. Given the breadth and diversity of the existing IPV assistance programme literature available, a scoping review is the most appropriate synthesis technique to address our research objectives.

## 2. Methods

We followed the framework for scoping reviews proposed by Arksey and O’Malley (2005), and used an integrated research process to obtain knowledge user input throughout all six stages of the review’s methodology. Knowledge users are defined as those who are ‘likely to be able to use research results to make informed decisions about health policies, programmes and/or practices’ (Canadian Institutes of Health Research, 2015). A collaboration of physicians, HCPs, researchers, IPV advocates, and IPV victim representatives (see Acknowledgements) made up the knowledge users for our scoping review and directed our research goals and methodology.

### 2.1. Literature search strategy

We consulted with a biomedical librarian to develop a sensitive search strategy to identify all types of publications involving IPV identification, assistance, and educational programmes in health care settings within the published literature. Several search strategies and sources were used to identify relevant studies. We used a combination of keywords and medical subject heading (MeSH) terms related to IPV, to search the following electronic databases: MEDLINE, Embase, Cumulative Index of Nursing and Allied Health Literature (CINAHL), Cochrane Database of Systematic Reviews (CDSR), Cochrane Central Register of Controlled Trials (CENTRAL), and psycINFO. All searches were performed in July 2015 and the search was limited to articles published from 2000 and onwards. No language restrictions were employed. Additionally, we conducted a hand search of systematic reviews, meta-analyses, and recently published included studies. A sample of the electronic search strategy is outlined in Appendix 1.

### 2.2. Eligibility criteria

We included studies in this scoping review if they met the following broad eligibility criteria: (1) published in English; (2) published in full-text format; (3) focused on IPV; (4) evaluated the effectiveness of an IPV assistance programme for women in a health care setting; (5) level I to IV evidence or used qualitative research methodology; and (6) population comprised of adults. Systematic reviews and meta-analyses were included if they otherwise met our eligibility criteria. We excluded studies that described an IPV screening programme but did not evaluate its effectiveness. We also excluded narrative reviews and studies that were published as dissertation abstracts or conference proceedings.

### 2.3. Article selection

We reviewed titles of all references identified in the literature search independently and in duplicate. We also reviewed abstracts of all references identified as potentially eligible during title screening independently and in duplicate. During title screening and abstract screening, reviewers erred on the side of inclusion and included any title that may have potentially met the eligibility criteria. Any conflicts between reviewers about whether a title or abstract was potentially eligible resulted in inclusion at this stage of the selection process. Two reviewers independently reviewed the full-text articles of all references included at the abstract screening level. Any conflicts between the two reviewers were discussed until consensus was reached. All article screening was completed using the web-based program DistillerSR.

### 2.4. Data extraction

We completed data extraction for all included studies independently and in duplicate. Any disagreements in data extraction were resolved by a third reviewer. We completed data extraction in DistillerSR using pre-designed data extraction forms which were piloted to ensure all key information was captured. We provided an instruction manual to each reviewer detailing instructions for data extraction to ensure consistency and accuracy of the extracted data.

Briefly, we extracted data related to study characteristics (e.g. location of research, year of publication, type of journal, etc.), study design characteristics (e.g. study design, number of participants, etc.), methodological characteristics (e.g. use of control group, follow-up, drop-out rate, etc.), programme evaluation (i.e. categories of outcome measures), and assistance programme characteristics (e.g. type of assistance provided, HCP delivering assistance, number of intervention sessions, etc.). Types of assistance programmes were classified as either active or passive. Active assistance included counselling/advocacy, home visitation, referral, safety assessment/planning, case management, and system change interventions. Passive assistance included providing IPV resources, videos, and provider cueing. Based on these categorizations, programmes were classified as either including a single type of active assistance, a single type of passive assistance, multiple types of active assistance, multiple types of passive assistance, or a combination of passive and active assistance. Additionally, studies were classified based on programme effectiveness as determined by author conclusions (i.e. positive versus not positive).

### 2.5. Data analysis

As per scoping review methodology, we used descriptive statistics to summarize all data. For continuous data, we reported the mean and standard deviation or median and interquartile range (IQR) based on the distribution of the data. We used counts and proportions to describe all other data. No inferential statistical testing was performed.

## 3. Results

**Figure 1.**
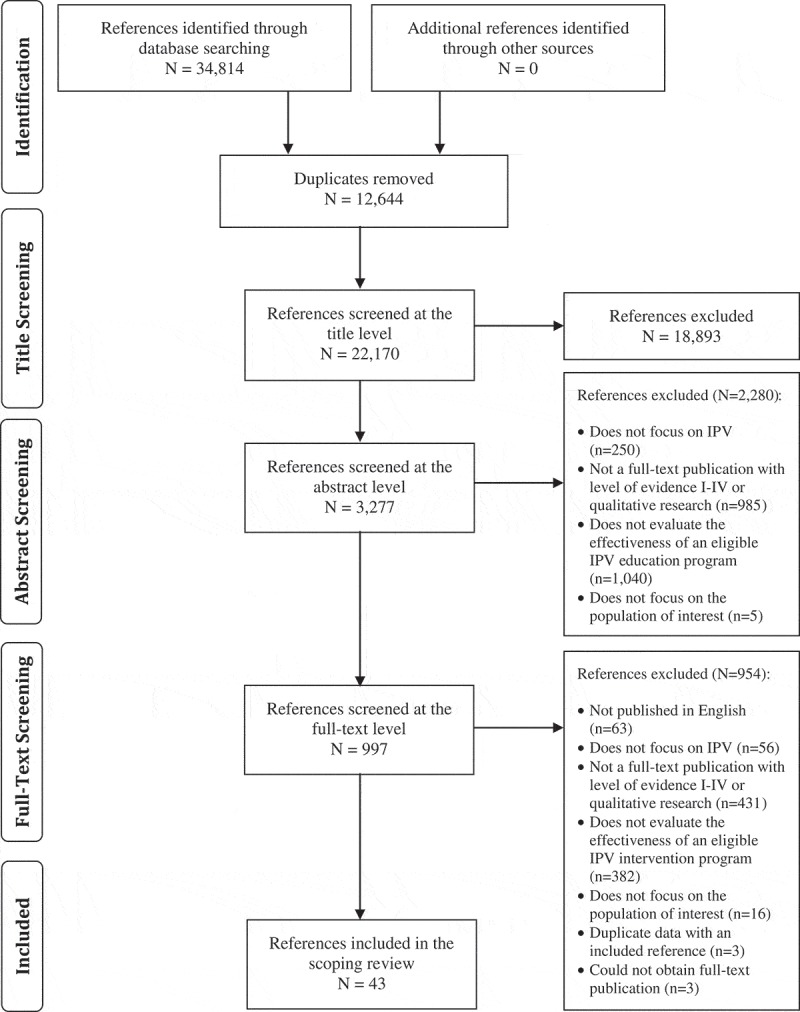
Literature search results and study selection.

Our literature search identified 22,170 unique references (Figure 1). A total of 21,173 references were excluded during title and abstract screening leaving 997 articles eligible for full-text review. Of the 997 articles that were reviewed at the full text level, 43 met the eligibility criteria and were included in our scoping review (Appendix 2).

### 3.1. Study characteristics

The majority of research took place in North America (*n* = 28, 65.1%), Europe (*n* = 7, 16.3%), and Australia/Oceania (*n* = 5, 11.6%) (Table 1). Only five studies were conducted in South America, Africa, and Asia combined. Approximately two-thirds of the publications (*n* = 26, 60.5%) occurred between the years 2010 and 2015 compared to 21% (*n* = 9) between 2000 and 2004 and 19% (*n* = 8) between 2005 and 2009. Studies were most commonly published in medical journals (*n* = 20, 46.5%) and women’s health or IPV journals (*n* = 13, 30.2%). The majority of included studies were funded (*n* = 34, 79.1%). Funders were typically governments (*n* = 16, 47.1%) or a combination of government and not-for-profit agency (*n* = 11, 32.4%).

**Table 1.** Study characteristics.

	Frequency
Characteristic	*N* (%) *N* = 43
*Location of Research*^a^:	
North America	28 (65.1)
Europe	7 (16.3)
Australia/Oceania	5 (11.6)
South America	2 (4.7)
Africa	2 (4.7)
Asia	1 (2.3)
*Year of Publication:*	
2000–2004	9 (20.9)
2005–2009	8 (18.6)
2010–2015	26 (60.5)
*Type of Journal:*	
Medical Journal	20 (46.5)
Women’s Health or IPV Journal	13 (30.2)
Nursing Journal	5 (11.6)
Multidisciplinary Journal	2 (4.7)
Social Science Journal	1 (2.3)
Health Services/Health Policy Journal	1 (2.3)
Systematic Review Journal	1 (2.3)
*Study Funding:*	
Funded (*n* = 34)	34 (79.1)
Government	16 (47.1)
Government and Foundation/Association/Non-Profit	11 (32.4)
Foundation/Association/Non-Profit	6 (17.6)
Foundation/Association/Non-Profit + Industry/Corporate/Profit	1 (2.9)
Not Specified	7 (16.3)
Unfunded	2 (4.7)

^a^Numbers do not sum to 43 and percentages do not sum to 100 as two studies were conducted in multiple locations.

### 3.2. Study design and methodological characteristics

Nearly half of the included studies were randomized controlled trials (*n* = 19, 44.2%) (Table 2). Other study designs included qualitative (*n* = 5, 11.6%), prospective comparative (*n* = 4, 9.3%), pre-test/post-test (*n* = 4, 9.3%), systematic reviews (*n* = 4, 9.3%), mixed methods (*n* = 3, 7.0%), case series (*n* = 3, 7.0%), and cross sectional (*n* = 1, 2.3%). Studies were conducted in a variety of health care settings with family medicine being the most frequent (*n* = 18, 41.9%), followed by obstetrics/gynaecology (*n* = 9, 20.9%), and emergency departments (*n* = 7, 16.3%). The majority of studies included more than one participating centre (*n* = 26, 70.3%).

**Table 2.** Study design and methodological characteristics.

	Frequency
Characteristic	*N* (%) *N* = 43
*Study Design:*	
Randomized Controlled Trial	19 (44.2)
Qualitative Study	5 (11.6)
Prospective Comparative Study	4 (9.3)
Pre-Test/Post-Test	4 (9.3)
Systematic Review	4 (9.3)
Mixed-Methods Study	3 (7.0)
Case Series	3 (7.0)
Cross-Sectional Study	1 (2.3)
*Number of Centres (n = 37)*^a^:	
1	11 (29.7)
2–4	11 (29.7)
5–9	8 (21.6)
≥10	7 (18.9)
*Study Setting*^b^:	
Family Medicine	18 (41.9)
Obstetrics/Gynaecology	9 (20.9)
Emergency Department	7 (16.3)
Home Visiting Programme	5 (11.6)
Prenatal/Antenatal Clinic	4 (9.3)
Paediatrics	3 (7.0)
Family Planning Clinic	3 (7.0)
Internal Medicine	2 (4.7)
*Victim Identification Method*^c^:	
Universal Screening	23 (59.0)
Targeted Screening	7 (17.9)
Referral from Another Service	5 (12.8)
Not specified	4 (10.3)
*Number of Participants (median [1^st^ Q, 3^rd^ Q])*	239 (64, 584)
*Age of Participants (mean, SD)*	30.3 (6.3)
*Use of Control/Comparative Group (n = 39)*^c^	30 (76.9)
*Inclusion of a Follow-Up Period (n = 39)*^c^	33 (84.6)
*Length of Follow-Up (months) (n =* 33):	
<0 to ≥3	8 (24.2)
<3 to ≥6	3 (9.1)
<6 to ≥12	4 (12.1)
<12	9 (27.3)
Not Reported	9 (27.3)
*Dropout Rate (n =* 33):	
0	0 (0.0)
1–10	3 (9.1)
11–20	3 (9.1)
21–30	6 (18.2)
>30	10 (30.3)
Not Reported	11 (33.3)
*Categories of Outcome Measures* (*n* = 39)^b,c^:	
IPV Severity/Recurrence	21 (53.8)
Use of IPV Resources	16 (41.0)
Mental Health/Wellbeing	13 (33.3)
Safety	12 (30.8)
Patient Opinions Towards Programme	11 (28.2)
Health Care Provider Opinions about the Programme	10 (25.6)
IPV Screening/Identification/Disclosure	9 (23.1)
IPV Discussions with Health Care Provider	7 (17.9)
Referral Rate to IPV Services	6 (15.4)
Physical Health/Wellbeing	6 (15.4)
Health Care Provider Knowledge/Attitudes/Self-Efficacy	5 (12.8)
Use of Non-IPV Related Healthcare Resources	4 (10.3)
Social Support	4 (10.3)
Stage of Change	3 (7.7)
Programme Harm Measures	3 (7.7)
Substance Use/Abuse	3 (7.7)
Community Involvement/Connection	3 (7.7)
Barriers to HCP Assistance Provision	3 (7.7)
Quality of Life	2 (5.1)
Women’s Relationship Statuses	2 (5.1)
Children’s Health	2 (5.1)
Economic/Efficiency	1 (2.6)
Parenting	1 (2.6)
Victim Self-Efficacy	1 (2.6)

^a^Number of centres was either not applicable or not reported for six studies.

^b^Numbers do not sum to 43 and percentages do not sum to 100 as some studies reported multiple characteristics.

^c^Data not abstracted for four systematic reviews.

The average age of women participating in the included studies was 30.3 years (*SD* = 6.3) (Table 2). The median number of participants across all studies was 239 (1^st^ Q = 64, 3^rd^ Q = 584). The average number of participants was 3745.5 (*SD* = 19,471.4) which was influenced by a large outlier from a large clustered randomized controlled trial that included 125,155 participants (Taft et al., 2015). When this outlier was excluded, the average number of participants was 710.3 (*SD* = 1204.8).

The majority of studies included a control or comparative group (*n* = 20, 76.9%) and a follow-up period (*n* = 33, 84.6%) (Table 2). The length of follow-up periods varied across studies with approximately one-quarter of studies reporting follow-up periods over one year (*n* = 9, 27.3) and under three months (*n* = 8, 24.2). Approximately 10% of studies reported follow-up periods of 3–6 months (*n* = 3, 9.1%) or 6–12 months (*n* = 4, 12.1%). One-quarter of studies did not clearly report their length of follow-up (*n* = 9, 27.3%). Approximately one-third of studies reported drop-out rates above 30% (*n* = 10, 30.3%) and under 30% (*n* = 12, 36.4%). Eleven studies (33.3%) did not report their drop-out rate.

### 3.3. Categories of outcome measures

A variety of categories of outcome measures were used to evaluate the IPV assistance interventions (Table 2). The most commonly used category of outcome measure was IPV severity/recurrence (*n* = 21, 53.8%) followed by use of IPV resources (*n* = 16, 41.0%), and mental health/wellbeing (*n* = 13, 33.3%). Twenty-one additional categories of outcome measures were reported in less than one-third of the included studies.

### 3.4. Types of assistance programmes

IPV victims were identified for inclusion in assistance programmes through universal screening (*n* = 23, 59.0%), targeted screening (*n* = 7, 17.9%), and referral from outside services (*n* = 5, 12.8%) (Table 3). Nine categories of assistance programmes were identified which included: counselling/advocacy, safety assessment/planning, referral, providing IPV resources (e.g. brochures, posters, etc.), home visitation, case management, videos, provider cueing, and system changes (Table 4). The most common type of assistance was counselling/advocacy (*n* = 25, 64.1%), followed by referral (*n* = 19, 48.7%), and safety assessment/planning (*n* = 15, 38.5%). The majority of studies evaluated programmes that included more than one type of assistance (*n* = 28, 73.7%). Programmes that included multiple active interventions were the most frequently evaluated (*n* = 16, 41.0%).

**Table 3.** Intervention characteristics.

		Frequency	
		Total (%)	
		Programme Effectiveness^b^	
		Positive	Not Positive
		*N* (%)	*N* (%)
Characteristic	Total *N* (%) *N* = 39^a^	*N* = 23 (59.0)	*N* = 16 (41.0)
*Type of Assistance Provided*			
Passive Assistance Alone	2 (5.1)	1 (50.0)	1 (50.0)
Active Assistance Alone	9 (23.1)	7 (77.8)	2 (22.2)
Multiple Passive Types of Assistance	1 (2.6)	1 (100.0)	0 (0.0)
Multiple Active Types of Assistance	16 (41.0)	9 (56.3)	7 (43.7)
Combination of Passive and Active Assistance	11 (28.2)	5 (45.5)	6 (54.5)
*HCP Delivering Intervention:*			
Nurse	10 (25.6)	4 (40.0)	6 (60.0)
Social Worker	7 (17.9)	4 (57.1)	3 (42.9)
Physician/Surgeon	6 (15.4)	2 (33.3)	4 (66.7)
Counsellor/Community Worker/Case Manager	6 (15.4)	5 (83.3.)	1 (16.7)
Non-Health Care Professional	6 (15.4)	4 (66.7)	2 (33.3)
Physician/Medical Assistant	4 (10.3)	2 (50.0)	2 (50.0)
IPV advocate/champion/coordinator	3 (7.7)	1 (33.3)	2 (66.7)
Nurse Practitioner	2 (5.1)	1 (50.0)	1 (50.0)
Computer	2 (5.1)	2 (100.0)	0 (0.0)
Passive (e.g. posters, brochures, etc.)	2 (5.1)	1 (50.0)	1 (50.0)
Other	4 (10.3)	4 (100.0)	0 (0.0)
Not Specified	3 (7.7)	1 (33.3)	2 (66.7)
*Number of Intervention sessions (mean, SD)*			
1	7 (17.9)	5 (71.4)	2 (28.6)
2	2 (5.1)	1 (50.0)	1 (50.0)
3	1 (2.6)	1 (100.0)	0 (0.0)
4	2 (5.1)	1 (50.0)	1 (50.0)
5	1 (2.6)	0 (0.0)	1 (100.0)
>5	5 (12.8)	5 (100.0)	0 (0.0)
Not Reported	21 (53.8)	10 (47.6)	11 (52.4)
*Total Length of Intervention in Hours (mean, SD)*			
<1	5 (12.8)	3 (60.0)	2 (40.0)
≥1 to <2	2 (5.1)	1 (50.0)	1 (50.0)
≥2	1 (2.6)	0 (0.0)	1 (100.0)
Not Reported	31 (79.5)	19 (61.3)	12 (38.7)
*Assistance Training Provided to HCPs*	29 (74.4)	17 (58.6)	12 (41.4)

^a^Data not abstracted for four systematic reviews.

^b^Programme effectiveness was abstracted based on study conclusions.

**Table 4.** Types of assistance programmes.

	Total
Type of Assistance Provided	*N* (%) *N* = 39^a^
Counselling/Advocacy	4 (10.3)
Counselling/Advocacy, Referral, and Safety Planning/Assessment	4 (10.3)
Referral	3 (7.7)
Systems Level Intervention	2 (5.1)
Counselling/Advocacy and Home Visitation	2 (5.1)
Counselling/Advocacy and IPV Resources (posters, pamphlets)	2 (5.1)
Counselling/Advocacy and Referral	2 (5.1)
Referral and Safety Planning/Assessment	2 (5.1)
Counselling/Advocacy, IPV Resources (posters, pamphlets) and Safety Planning/Assessment	2 (5.1)
Counselling/Advocacy, IPV Resources (posters, pamphlets), Referral, Safety Planning/Assessment and Systems Level Intervention	2 (5.1)
Provider Cueing	1 (2.6)
IPV Resources (posters, pamphlets)	1 (2.6)
Case Management and IPV Resources (posters, pamphlets)	1 (2.6)
Case Management and Video	1 (2.6)
Counselling/Advocacy and Safety Planning/Assessment	1 (2.6)
Referral and Home Visitation	1 (2.6)
Case Management, IPV Resources (posters, pamphlets) and Referral	1 (2.6)
Counselling/Advocacy, Home Visitation and Referral	1 (2.6)
Counselling/Advocacy, Home Visitation and Safety Planning/Assessment	1 (2.6)
Counselling/Advocacy, IPV Resources (posters, pamphlets) and Systems Level Intervention	1 (2.6)
IPV Resources (posters, pamphlets), HCP Cueing and Video	1 (2.6)
Counselling/Advocacy, Home Visitation, Referral and Safety Planning/Assessment	1 (2.6)
Counselling/Advocacy, Referral, Safety Planning/Assessment and Systems Intervention	1 (2.6)
Counselling/Advocacy, Referral, IPV Resources (posters, pamphlets), Safety Planning/Assessment and Case Management	1 (2.6)

^a^Data not abstracted for four systematic reviews.

A variety of different health care professionals delivered the assistance intervention including nurses (*n* = 10, 25.6%), social workers (*n* = 7, 17.9%), physicians/surgeons (*n* = 6, 15.4%), and counsellors, community workers, or case managers (*n* = 6, 15.4%) (Table 3). Approximately three-quarters of studies (*n* = 29, 74.4%) specified that training was provided to those delivering the intervention. Administering the intervention over one session was reported most frequently (*n* = 7, 17.9%), followed by five or more sessions (*n* = 5, 12.8%), however, approximately half of all studies did not report the number of intervention sessions (*n* = 21, 53.8%). The majority of studies also did not report the length of time of the intervention (*n* = 31, 79.5%), however, of the studies that did, most were less than an hour in length (*n* = 5, 12.8%).

### 3.5. Characteristics of effective programmes

We identified 23 studies (59.0%) that reported positive programme effectiveness, 15 studies (38.5%) that reported neutral or mixed programme effectiveness, and one study (2.6%) where the no conclusion was reached regarding programme effectiveness (Table 3). No studies were identified that reported negative programme effectiveness (i.e. programmes in which the results favoured the control group or found the intervention to be predominately harmful). We looked at intervention characteristics by stratifying all studies by programme effectiveness (i.e. positive versus not positive). Characteristics of programmes amongst studies frequently reporting positive results (i.e. positive results reported for ≥75% of studies in which at least four studies include the specified programme characteristic) included those in which one type of active assistance was used (77.8% of studies reported positive results), a counsellor, community worker, or case manager provided the intervention (83.3% of studies reported positive results) and programmes that were delivered over more than five sessions (100.0% of studies reported positive results).

We also looked at this in the subset of 21 studies that included IPV severity/recurrence as an outcome measure. Twelve of these studies (57.1%) reported positive programme effectiveness and nine (42.9%) reported neutral or mixed programme effectiveness (Table 5). Characteristics of programmes amongst studies frequently reporting positive results included those in which a counsellor, community worker, or case manager provided the intervention (80.0% of studies reported positive results) and programmes that were delivered over more than five sessions (100.0% of studies reported positive results).

**Table 5.** Intervention characteristics for studies reporting IPV severity/recurrence outcomes.

		Frequency	
		Total (%)	
		Programme Effectiveness^a^	
		Positive	Not Positive
		*N* (%)	*N* (%)
Characteristic	Total *N* (%) *N* = 21	*N* = 12 (57.1)	*N* = 9 (42.9)
*Type of Assistance Provided*			
Passive Assistance Alone	0 (0.0)	0 (0.0)	0 (0.0)
Active Assistance Alone	4 (19.0)	2 (50.0)	2 (50.0)
Multiple Passive Types of Assistance	0 (0.0)	0 (0.0)	0 (0.0)
Multiple Active Types of Assistance	10 (47.6)	6 (60.0)	4 (40.0)
Combination of Passive and Active Assistance	7 (33.3)	4 (57.1)	3 (42.9)
*HCP Delivering Intervention:*			
Counsellor/Community Worker/Case Manager	5 (23.8)	4 (80.0)	1 (20.0)
Nurse	4 (19.0)	1 (25.0)	3 (75.0)
Non-Health Care Professional	4 (19.0)	2 (50.0)	2 (50.0)
Social Worker	3 (14.3)	2 (66.7)	1 (33.3)
Physician/Surgeon	2 (9.5)	0 (0.0)	2 (100.0)
Physician/Medical Assistant	2 (9.5)	1 (50.0)	1 (50.0)
IPV advocate/champion/coordinator	1 (4.8)	0 (0.0)	1 (100.0)
Nurse Practitioner	1 (4.8)	1 (100.0)	0 (0.0)
Computer	1 (4.8)	1 (100.0)	0 (0.0)
Other	3 (14.3)	3 (100.0)	0 (0.0)
Not Specified	1 (4.8)	0 (0.0)	1 (100.0)
*Number of Intervention sessions (mean, SD)*			
1	5 (23.8)	3 (60.0)	2 (40.0)
2	0 (0.0)	0 (0.0)	0 (0.0)
3	1 (4.8)	1 (100.0)	0 (0.0)
4	0 (0.0)	0 (0.0)	0 (0.0)
5	1 (4.8)	0 (0.0)	1 (100.0)
>5	4 (19.0)	4 (100.0)	0 (0.0)
Not Reported	10 (47.6)	4 (40.0)	6 (60.0)
*Total Length of Intervention in Hours (mean, SD)*			
<1	4 (19.0)	2 (50.0)	2 (50.0)
≥1 to <2	0 (0.0)	0 (0.0)	0 (0.0)
≥2	1 (4.8)	0 (0.0)	1 (100.0)
Not Reported	16 (76.2)	10 (62.5)	6 (37.5)
*Assistance Training Provided to HCPs*	15 (71.4)	9 (60.0)	6 (40.0)

^a^Programme effectiveness was abstracted based on study conclusions.

## 4. Conclusions

Our study is the first scoping review to be conducted on the IPV assistance programme literature and represents a comprehensive overview of the available evidence. Our scoping review includes 43 studies evaluating many different programmes conducted in a variety of health care settings. We found that these types of studies are being conducted in all continents, however the majority of research is produced in North America (65%), Europe (16%), and Australia/Oceania (12%). South America, Africa, and Asia produced less than 5% each of the studies included in our scoping review. This may be indicative of differing attitudes towards IPV assistance interventions or research evaluating them in different parts of the world. We also found evidence that suggests interest in evaluating IPV assistance studies has increased over time, as over half of the studies included in our review were published between 2010 and 2015.

Nearly half of all studies included in our scoping review were randomized controlled trials. The majority of studies took place in 1–4 centres (59%) and few studies (19%) took place in 10 or more centres. The length of follow-up varied substantially across studies with only one-quarter of studies following participants for over a year. There was also substantial variability reported in drop-out rate with 37% of studies reporting drop-out rates of over 20% indicating the potential for threats to internal validity (Sacket, Richardson, & Rosenberg, 1997). High-quality randomized controlled trials are considered the highest level of evidence (Centre for Evidence-Based Medicine, 2009), however, it is important that trials ensure adequate length of follow-up in order to capture important changes in outcomes while also minimizing the drop-out rate in order to reduce bias. Future research should focus on conducting high-quality randomized controlled trials that include multiple centres and a large number of participants to achieve adequate power and generalizability of results.

The studies included in our scoping review used 23 different categories of outcome measures to evaluate programme effectiveness indicating a large amount of heterogeneity for outcome assessment. The most common category of outcome measure identified was IPV severity/recurrence which was included in 54% of studies. This category of outcome measure is consistent with the goal of many IPV assistance programmes of reducing further abuse (Jahanfar et al., 2014). Interestingly, only programmes that included active assistance (either alone or combined with other passive or active types of assistance) included this category of outcome measure. No studies evaluating solely passive assistance interventions that included IPV severity/recurrence as a category of outcome measure were identified. This may be due to a belief that more active interventions are required in order to affect change in this outcome measure. The heterogeneity in outcome assessments creates challenges for comparing results between studies and pooling studies for meta-analysis. This is highlighted by the finding that none of the four meta-analyses that have been conducted in the area of IPV assistance programmes were able to pool outcome data due to heterogeneity (Bair-Merritt et al., 2014; Jahanfar et al., 2014; O’Reilly, Beale, & Gillies, 2010; Van Parys et al., 2014). Future research should focus on identifying the most appropriate outcome measures and conducting high-quality randomized controlled trials using these outcomes to facilitate the conduct of systematic reviews and meta-analyses.

Our scoping review identified nine categories of IPV assistance (counselling/advocacy, safety assessment/planning, referral, providing IPV resources [e.g. brochures, posters, etc.], home visitation, case management, videos, provider cueing, and system changes). IPV assistance programmes included in the study were heterogeneous and 24 different combinations of assistance categories were used. Interventions ranged in intensity from programmes that included only one form of passive assistance (e.g. provider cueing or IPV resources only) to programmes that included system level interventions. For example, Calderón, Gilbert, Jackson, Kohn, and Gerbert (2008) examined the effects of an HCP cueing intervention that involved attaching a ‘cueing sheet’ to patient’s medical record at the time of their appointment. The cueing sheet provided HCPs with the patient’s IPV screening results and suggested possible counseling statements. Another study conducted by Bair-Merritt, Mollen, You, and Fein (2006) examined women’s opinions about an IPV resource intervention that involved displaying posters and cards arounds a paediatric emergency department for a 24-hour IPV helpline. This is in contrast to other studies that have implemented system level interventions which involve multiple interventions (McCaw, Berman, Syme, & Hunkeler, 2001; McNutt, Carlson, Rose, & Robinson, 2002). System level interventions include multiple IPV interventions that are delivered at different levels such as environmental changes (e.g. displaying IPV resources, identifying private space to discuss IPV with patients), local procedure changes (e.g. screening and referral protocols and pathways), staff changes (e.g. education for staff, intensive training for select staff members to become IPV experts, employment of IPV experts), service changes (e.g. adoption of onsite IPV services), community engagement (e.g. increased collaboration with community-based IPV services).

The majority of studies identified by our scoping review (59%) reported positive programme evaluation results. The other 41% reported neutral, mixed, or inconclusive results and no studies reported negative results (i.e. programmes in which the results favoured the control group or found the intervention to be predominately harmful). This finding may suggest that IPV assistance programmes are unlikely to be of more harm than benefit to women who receive them. Alternatively, it could represent a bias in the literature such as publication or outcome selection bias. It is also possible that programme harm was not adequately evaluated or reported in many studies. Our scoping review found only three studies that reported programme harm measures (Hegarty et al., 2013; McFarlane, Groff, O’Brien, & Watson, 2006; Tiwari et al., 2005); all three studies were randomized controlled trials which monitored participants for adverse events. No adverse events were identified by any of the three studies. While only three studies reported the inclusion of adverse events as an outcome measures and presented the results, it is possible that other studies also collected these data but did not report the results for this outcome. Additionally, one study (Hegarty et al., 2013) reported results pertaining to awareness of participants’ partners about their participation in the trial and their reactions. At the time of final follow-up, they found that 28% of participants in the intervention group had partners who were aware of their study participation compared to 13% of participants in the control group. For both the intervention and control group, less than 1% of participants reported positive partner reactions (e.g. improved partner behaviour and attempts to change IPV behaviour) and less than 0.5% reported negative reactions (e.g. anger, or behaviours that illicit fear in the participant or restrict her freedom). There were no significant differences in partners’ positive or negative reactions between the intervention and control group. Reporting programme harm measures such as adverse events and partner’s awareness and reactions to study participation is an important component of assessing the benefits and risks of interventional programmes for knowledge users and the consort checklist for non-pharmacologic treatment interventions recommends reporting all important adverse events (Boutron, Moher, Altman, Schulz, & Ravaud, 2008). Future research should ensure that data are collected and reported for any adverse events possibly related to the intervention to allow for better assessment of any harms (or lack thereof) resulting from IPV assistance programmes.

It is surprising that studies that reported using only one type of active assistance reported positive programme evaluations more frequently than studies evaluating multiple types of programme assistance. This may be a spurious finding due to small sample size. It is also likely that studies vary in terms of the detail used to describe their interventions and that some studies that only mentioned one type of assistance actually included multiple types of assistance. For example, studies that just mentioned counselling interventions could have included referrals or safety planning within the counselling sessions but just not specified this level of intervention detail. Future research should ensure that IPV assistance programme interventions are reported in adequate detail to allow for their replication. Reporting adequate intervention details will also facilitate the conduct of future systematic reviews and meta-analyses.

Finally, our scoping review identified three studies (Loughlin, Spinola, Stewart, Fanslow, & Norton, 2000; McGarry & Nairn, 2015; Rees et al., 2014) that reported results pertaining to barriers HCPs experienced to providing IPV assistance. These studies identified barriers at both the infrastructure level (e.g. staff time, availability of private locations within health care settings, inadequate follow-up resources, and availability of referral services) and the HCP level (e.g. concerns about offending patients, perceptions of inadequate knowledge and skills to provide assistance, and attitudes towards IPV and HCPs role in assisting with it). Similar barriers have been reported in other studies examining barriers to IPV screening and assistance within health care settings (Gutmanis, Beynon, Tutty, Wathen, & MacMillan, 2007; Jaffee, Epling, Grant, Ghandour, & Callendar, 2005; Sprague et al., 2012, 2013). Future research should focus on ways to address these barriers in order to promote the implementation of effective IPV assistance programmes within health care settings.

Several strengths contribute to the quality of this scoping review. Our broad search strategy was designed and conducted by a research librarian with expertise in the area to ensure all published literature was captured. Additionally, all article screening and data extraction was completed in duplicate by reviewers with both content and methodological expertise. Finally, broad eligibility criteria were used allowing our scoping review to capture any type of IPV assistance programme delivered within a health care setting. To date no scoping reviews have been conducted in this area and only four systematic reviews have been conducted. Of the four systematic reviews, three focused solely on interventions for pregnant women (Jahanfar et al., 2014; O’Reilly et al., 2010; Van Parys et al., 2014) and one focused solely on assistance interventions that took place within primary care (Bair-Merritt et al., 2014).

Despite these strengths, our scoping review is limited by the restriction of our search to published literature. This may introduce publication bias into our results as it is possible that there are a higher proportion of studies with neutral or negative results that are unpublished compared to positive ones. Our scoping review was also limited to studies published in English which may partially explain the limited number of studies found that were conducted in Asia, South America, and Africa. Additionally, the heterogeneity of study design and interventions introduced challenges of capturing all of the details of each included study. However, by focusing our scoping review on the commonalities between studies we were able to produce a comprehensive overview of the existing literature.

Overall, our scoping review provides a comprehensive summary of the literature evaluating IPV assistance programmes in health care settings. Our results demonstrate that IPV assistance programmes are heterogeneous with regard to the types of assistance they include and how they are delivered and evaluated. This heterogeneity creates challenges in identifying which IPV assistance programmes, and which aspects of these programmes, are effective. However, it appears that many different types of IPV assistance programmes can have positive impacts on women. More high-quality research is needed in order to make definitive conclusions and clear practice recommendations.

## Highlights of the article

- A total of 43 studies were published between 2000 and 2015 that looked at programmes in health care settings designed to help women who are victims of intimate partner violence.
- These programmes included many kinds of help for women including counselling, referral, safety planning, and providing resources.
- Most of these programmes (59%) were found to be beneficial to women.

## Appendices Appendix 1.Search strategy (e.g. MEDLINE)

Database: Ovid MEDLINE(R) In-Process & Other Non-Indexed Citations, Ovid MEDLINE(R) Daily and Ovid MEDLINE(R) <1946 to Present>

Search Strategy:

spouse abuse.mp. or exp Spouse Abuse/

(intimate partner adj4 (abus* or aggression or sexual or violence or victimization or victim*)).mp.

(partner adj4 (abus* or aggression or violence or victimization or victim*)).mp. (5153)

(domestic partner adj4 (abus* or aggression or sexual or violence or victimization or victim*)).mp.

(intimate adj2 (violence or victim:)).mp.

(woman abuse or battered spouse).mp.

(spousal adj (abuse or violence)).mp.

(woman abuse or battered spouse or battered woman).mp.

((wife or spouse or spousal or partner) adj battering).mp.

wife rape.mp.

((spousal or spouse) adj rape).mp.

batterer.mp.

((spouse or domestic or wife or spousal or woman) adj (abuse or abuser)).mp.

couple violence.mp.

or/1–14

battered women.mp. or Battered Women/

battering.mp.

domestic violence.mp. or Domestic Violence/

family violence.tw.

violence against women.mp.

or/16–20

((risk or experienc:) adj3 (abuse or violence: or abusive)).mp.

Mass Screening/

Risk Assessment/

health facilities/or exp academic medical centers/or ambulatory care facilities/or exp outpatient clinics, hospital/or pain clinics/or surgicenters/

Emergency Service, Hospital/or exp hospitals/

education.ti,kw.

(perpetrator: or abuser:).mp.

‘interprofessional education’.kw.

(assessment or screening).ti,kw.

training.mp.

hospital*.ti.

or/22–32

21 and 33

15 or 34

limit 35 to yr = ‘2000 -Current’
